# Empathy and Cognitive Processing as Factors Determining the Consequences of Secondary Exposure to Trauma Among Roman Catholic Clergymen

**DOI:** 10.1007/s10943-021-01443-y

**Published:** 2021-10-25

**Authors:** Zygfryd Juczyński, Nina Ogińska-Bulik, Józef Binnebesel

**Affiliations:** 1 Social Academy of Sciences, Tokarzewskiego 2, 91-842 Łódź, Poland; 2 University of Łódź, Łódź, Poland; 3 Nicolaus Copernicus University, Toruń, Poland

**Keywords:** Clergymen, Secondary traumatic stress, Vicarious posttraumatic growth, Empathy, Cognitive processing

## Abstract

By helping individuals after traumatic experiences, the helper is also exposed to the consequences of trauma. The aim of this study was to determine the predictors of consequences of secondary exposure to trauma among clergymen and therapists (*N* = 140) helping victims of trauma in Poland. Symptoms of secondary traumatic stress (STS) were identified in 22.9% of the clergymen and 8.6% of therapists, and secondary posttraumatic growth (SPTG) in 61.4% of the clergymen and 42.9% of therapists. STS and SPTG predictors were identified based on regression models. In the case of clergymen, regret is a determinant of STS, and positive cognitive restructuring of SPTG. Research findings highlight the need to increase awareness of exposure to secondary trauma among the clergy, and for them to acquire greater skills for coping with stress.

## Introduction

### Two Faces of Secondary Traumatization

Many people experience traumatic events in their lives, ranging from injuries and traffic accidents to violence and life-threatening disease. In such situations, they usually seek help from the emergency services, and over a longer period, by professionals such as therapists, social workers, probation officers, or carers. For many sufferers of trauma, a significant source of moral support is the clergyman.

Studies suggest that helpers may also experience changes in their psyche and behavior by providing constant help, empathic engagement, and support. The earliest findings, dating from the 1970s, reported the occurrence of burnout in the nursing profession. Later studies found those in the caring professions to be more susceptible to a unique form of burnout, known as *compassion fatigue* (Abendroth, 2011). McCann and Pearlman (1990) found helpers to significantly change or reform their beliefs concerning themselves and the world after working with trauma survivors; these changes, later defined as *vicarious traumatization*, appeared to arise because of empathic involvement with those who had experienced trauma. Such changes usually developed over many years of working with traumatized clients or patients, and often had long-lasting effects. Figley (1995) coined this state of indirect traumatic experience as *secondary traumatic stress* (STS) and defined the symptoms as “the natural consequent behaviours and emotions resulting from knowing about a traumatizing event experienced … by a person” (p. 7). These symptoms are almost identical to posttraumatic stress disorder (PTSD), which is known to occur following direct exposure to traumatic experience, and is characterized by intrusion, avoidance, negative changes in cognition or mood and increased arousal and reactivity.

STS is commonly observed among various professions working with trauma survivors. It is particularly common among social workers (Manning-Jones et al., 2017; Ogińska-Bulik & Juczyński, 2020; Ogińska-Bulik et al., 2020) and medical personnel (Ogińska-Bulik et al., 2021).

A sizable body of research into secondary trauma has been performed on therapists, and most of the findings suggest it occurs at low or moderate intensity in this group. The prevalence has been found to range from 9% among studied therapists (Daniels, 2006), 8% of American therapists, where it occurred at high or very high intensity (Froman, 2014) to 14% of therapists working with trauma victims, among whom it was observed at high intensity (Ogińska-Bulik et al., 2020). This relatively low prevalence of high intensity STS among therapists can result from their expertise in dealing with stress, and hence from their qualifications and experience as therapists. Indeed, a study of five occupational groups recorded the lowest level of STS among therapists (7.5%) (Ogińska-Bulik & Juczyński, 2020).

Even though mental health typically falls within the professional domain of qualified specialists, clerics also represent an equally important source of advice and moral support, especially for believers in God. It has been demonstrated that in critical, difficult times, clergymen are often the first source of help for many people. Clergymen have played an important role providing support in crisis situations caused by natural disasters (Chinnici, 1985), terrorist attacks (Flannelly et al., 2005), violence, and other social crises (Everly, 2000). Following such crises and significant events, many people experiencing trauma are forced to seek, discover, and confront their spiritual beliefs. However, such profound needs require the clergyman to go beyond the moral support usually offered to parishioners, and thus become exposed to negative effects. In fact, the stress reported by clergymen is like that reported by professionals dealing with mental health (Holaday et al., 2001).

A key aspect of the everyday work of the clergyman is to act as an advisor. One study of a representative sample of the population of the USA found that a quarter of those suffering from mental distress and seeking help for it first turned to a clergyman (Lount & Hargie, 1997). Their findings revealed that pastoral activities in hospitals in New York and Connecticut involved solving a wide range of problems ranging from sorrow, death and dying, depression and alcohol and drugs, to domestic violence, severe mental disorders, HIV/AIDS, and suicide. It is worth noting that clergymen involved in pastoral activities felt more self-confident while dealing with death and dying compared to other problems (Moran et al., 2005). Galek et al. (2011) identified a high level of STS among chaplains, and report that the number of hours the chaplains spent per week with traumatized persons was positively related to the degree of STS.

However, many decades of research indicates that, together with its negative consequences, traumatic experience can also foster positive posttraumatic changes, reflected in *posttraumatic growth* (PTG). Similar changes may occur among people exposed to secondary trauma, a phenomenon defined as *vicarious posttraumatic growth* or *secondary posttraumatic growth* (SPTG). SPTG is defined as the experience of growth following indirect exposure to trauma; it is believed to relate to the perception of oneself, one’s relations with others, and one’s philosophy of life (Arnold et al., 2005). Helping traumatized people can lead to beneficial changes in the personality of the helper, such as increased sensitivity, compassion and insight, greater self-confidence, tolerance, and empathy: in fact, Arnold et al. (2005) emphasize that following their work with traumatized clients, some of the surveyed therapists began to appreciate their lives more and tried to draw more from them. Although some also noted an increased awareness of the negative consequences of this type of work, this awareness led to a deeper understanding the complexity of the entire spectrum of human functioning.

SPTG has also been recorded among healthcare professionals (Shiri et al., 2008), especially those employed in the social service sector and those dealing with mental health (Cieślak et al., 2016). In a study comparing five helping professions (Ogińska-Bulik & Juczyński, 2020), the greatest prevalence of high SPTG levels was observed among therapists (47.5%) and the least among social workers (25.3%). However, no such studies have been performed on the prevalence of SPTG among clergymen.

### The Mechanisms of Secondary Posttraumatic Changes—The Role of Empathy and Cognitive Processing

Although the mechanism of development of secondary traumatization remains poorly understood, a few theoretical models have been proposed, and these have been chosen as the basis of the present study. While PTSD models take a cognitive approach to understanding secondary exposure to trauma, the Trauma Transmission Model developed by Figley (Figley, 1995; Ludick & Figley, 2016), and Empathy-based Stress by Rauvola et al. (2019) emphasize the role of empathy in STS development. According to Figley (1995), increased empathy, expressed in a form of empathic concern, elicits empathic responses; over time, such constant empathic responses can have a numbing effect and elicit STS symptoms. While Ravuola et al. (2019) also highlights the role played by empathy, they also indicate that other factors can hasten the development of the negative effects of secondary exposure to trauma. They define such empathy-based stress “as a stressor–strain-based process of trauma at work, wherein exposure to secondary or indirect trauma, combined with empathic experience, results in empathy-based strain and additional outcomes (i.e., other occupational health/strain outcomes; work affect, behaviors, and cognitions)” (p. 299).

The role of cognitive activity in the development of both the negative and positive effects of secondary exposure to trauma is underlined by Cohen and Collens (2013). According to the authors, both behavioral and cognitive coping efforts are primarily related to the search for organizational support and the use of personal resources. In this sense, the cognitive activity undertaken in response to exposure to traumatic situations can account for its resulting negative and positive effects.

Tedeschi and Calhoun (2004) propose that the psychological processes accompanying the occurrence of negative effects of traumatic experience also lie at the core of posttraumatic growth, and that growth occurs in situations when individuals change their ways of perceiving themselves and the world. These changes rely on a deeper insight and an ability to give meaning. As a result, experiencing loss is transformed into an important value. Moreover, this deeper insight into oneself and the surrounding reality allows people to deal successfully with adversity in future; such growth not only allows them to survive such difficulties, but also to triumph over them.

Cohen and Collens (2013) outline that VPTG stemming from empathic engagement in helping leads to changes within the cognitive schemas of the helping professional. They emphasize that these changes are both positive and negative, and that the two can co-occur. This model illustrates VPTG as more than simply positive emotions, but rather a cognitive change conducive for self-actualization.

The above-mentioned conceptualizations emphasize the involvement of empathy and cognitive processing in the occurrence of the negative and positive effects of secondary exposure to trauma. Empathy can be regarded as having two components: the ability to perceive the emotional conditions of other people (emotional empathy), and the ability to adopt their way of perceiving reality (cognitive empathy). The former enables individuals to maintain emotional contacts with others, and then to respond emotionally to their suffering, while the latter allows them to understand a particular situation, and to behave in a way which expresses concern about others. It is important to distinguish between *knowing* that somebody is suffering (cognitive empathy) and *understanding* that someone is feeling that person’s pain (emotional empathy) (Bloom, 2017). While working with trauma survivors, the empathy felt by the helper may contain one or both above-mentioned elements; however, while the presence of emotional empathy has been associated with increased vulnerability to the symptoms of STS, the cognitive component is believed to increase the probability of SPTG.

The aim of the conducted research is to:

- identify the effects of secondary exposure to trauma in the form of secondary posttraumatic stress (STS) and growth (SPTG) among clergy and therapists,
- identify the relationship between empathy and coping strategies, and the consequences of secondary exposure to trauma in the form of STS and SPTG,
- determine the predictors of STS and SPTG in the compared groups.

## Method

### Sample and Procedure

The study comprised two groups. The first group comprised Roman Catholic clergymen helping, supporting, or accompanying people who had personally experienced traumatic events. The participants were providing pastoral care in hospitals, various specialist centers, hospices, health care centers, prisons, resocialization centers, and penitentiary institutions in Poland. They were supplied questionnaires, either in paper form or as a link to an electronic version, by persons coordinating their work, after obtaining the consent of superiors. The second group, treated as a comparative group, was composed of qualified psychotherapists, most of whom had completed a psychological education and had spent at least half of their working time helping trauma survivors.

In total, 70 correctly completed questionnaires were received from the clerics, 60% of which were online. Similarly, 70 completed questionnaires were received from therapists. The clergymen participating in the study were aged from 28 to 66 years old, and their mean length of professional practice was 18.80 years. The main differences between the compared professional groups concern gender. While all clergymen are men, almost 90% of the group of therapists are women. The main differences between the compared professional groups concern gender. While all clergymen are men, almost 90% of the group of therapists are women. More details are presented in Table 1.

**Table 1:** Baseline characteristics of respondents

Study groups		Clergymen	Therapeuts
*Sex*			
Men	N (%)	70 (100.0)	9 (12.9)
Women	N (%)	0 (0.0)	61 (87.1)
*Age*	Mean (SD)	46.19 (9.9)	37.47 (7.8)
	Range	28–66	26–58
Seniority (in years)	Mean (SD)	18.80 (9.9)	9.27 (6.4)
	Range	2–35	1–25
Personal trauma experiences	N (%)	39 (55.7)	16 (22.8)
Country participating in the study		Poland	Poland

All procedures performed in the study involving human participants were under the ethical standards of the Institutional Research Committee of the institutions employing the authors of the study. The survey was anonymous and did not require providing any personal data.

### Measures

The following measurement tools were used in the research:

**Secondary Traumatic Stress Inventory** (STSI) is a modification of the adapted PTSD Checklist, developed by Weathers et al. (2013). The STSI consists of 20 statements, evaluated on a five-point Likert scale, concerning assistance provided to people after traumatic experiences (Ogińska-Bulik et al., 2018). In addition to the overall result, the inventory includes four basic criteria for PTSD. Factor analysis (exploratory and confirmatory) confirmed the four-factor structure of the scale: Intrusion, Avoidance, Negative alterations in cognition and mood, and Alterations in arousal and reactivity. Internal consistency is good, with a Cronbach’s α score of 0.95. For differential diagnosis, a cut-off point of 33 points was determined.

**Secondary Posttraumatic Growth Inventory** (SPTGI), developed by Ogińska-Bulik and Juczyński (2020), is used to measure the positive changes deriving from secondary traumatic stress. It consists of 12 items, evaluated on a six-point scale. Factor analysis identified four factors: New challenges and competences, Changes in spirituality, Changes in self-perception and Increased acceptance and actions for other. The tool demonstrates satisfactory internal consistency for total score and stability (Cronbach's alpha coefficients 0.90; test–retest 0.78). A score of 38 points (lower limit of 7 sten) was set as the cut-off point for a high level of positive posttraumatic change. Raw scores are converted into units standardized on the sten scale.

**Empathetic Sensitivity Scale** (ESS) is a modification the Interpersonal Reactivity Index, based on Davis’s theory of empathy (Davis, 2006). The ESS contains 28 items evaluated on a five-point scale and measures three components. Based on an exploratory and confirmatory factor analysis, a three-factor model was adopted. The scale of Empathetic concern captures the tendency to be compassionate with people who have failed; the Personal distress scale measures the tendency to experience anxiety, distress, or discomfort in response to strong negative experiences of other people, and the Perspective taking scale measures the ability to "go beyond self" when communicating with other people. The first two refer to emotional empathy, and the third to the cognitive aspect (Kaźmierczak et al., 2007). Internal consistency is good, with a Cronbach’s α score of 0.74 to 0.78. Raw scores are converted into units standardized on the sten scale.

**Cognitive Processing of Trauma Scale** (CPOTS), originally developed by Williams et al. (2002). The Polish adaptation by Ogińska-Bulik and Juczyński (2018) consists of 17 items assessed on a seven-point scale. The CPOTS measures five strategies used to cope with STS. Three of these strategies reflect the positive processing of trauma (adaptive strategies): Cognitive restructuring refers to the ability to see the positive side of a negative event; Resolution/acceptance involves solving or accepting a problem, and Downward comparison attempts to regard the situation as being not as bad as that faced by other people. The remaining two reflect the negative processing of trauma (non-adaptive strategies): Regret, associated with thinking that nothing could be done and blaming oneself for what happened, and Denial, i.e., pretending it did not really happen. Factor analyzes confirmed that the tool has a five-factor structure, like that of original version, but with a changed order. The reliability and accuracy of the scale are satisfactory; Cronbach’s alpha coefficients for individual strategies range from 0.56 to 0.89.

### Data Analysis

Data analyses were carried out using the SPSS statistical package (version 20). The distributions of the variables were checked for normality based on skewness and kurtosis (values less than ± 1). No significant differences in the STS and SPTG scores were found between the “paper–pencil” test and the on-line survey. The Hedge's *g* statistic was used to measure the effect size for the difference between means (STS on-line: *M* = 23.00; SD = 14.01; “paper–pencil”: *M* = 21.86; SD = 13.65; *g* = 0.08; SPTG on-line: *M* = 41.80; SD = 9.45; “paper–pencil”: *M* = 38.14; SD = 9.27; *g* = 0.39). Descriptive statistics were calculated, and effect sizes for parametric data were calculated using Pearson’s correlation coefficient (*r*). The models were verified using linear regression analysis (step method).

## Results

### Descriptive Analysis—Comparison of Study Groups

The clergymen participating in the research demonstrated greater intensification of secondary traumatic changes than therapists (Table 2). Although this situation applies to both the negative and positive symptoms, more significant differences were noted for the negative ones. The analysis of variance indicated a greater intensification of avoidance symptoms among clergymen. In addition, regarding secondary posttraumatic growth (SPTG), the two groups demonstrated significant differences regarding changes in spirituality (*p* < 0.001).

**Table 2:** Descriptive analysis—Comparison of study groups

Variables	Clergymen (*N* = 70)		Therapists (*N* = 70)		*t*	*p*
	*M*	SD	*M*	SD		
Secondary Traumatic Stress—total score	22.43	13.98	12.34	11.84	4.61	< .001
Intrusion	1.08	0.89	0.68	0.62	3.08	< .01
Avoidance	1.31	1.00	0.49	0.63	5.81	< .001
Negative alterations in cognitions	1.07	0.73	0.56	0.59	4.49	< .001
Alterations in arousal and reactivity	1.16	0.90	0.67	0.81	3.35	< .001
Secondary Posttraumatic Growth—total score	39.97	9.36	32.26	12.48	− 4.14	< .001
New challenges and competences	3.32	0.96	2.95	1.15	2.05	< .05
Changes in spirituality	3.25	0.97	1.28	1.33	9.97	< .001
Changes in self-perception	3.67	1.44	3.31	1.35	1.49	ns
Increased acceptance and actions for other	3.09	0.96	3.20	1.37	− 0.57	ns
*Empathy:*						
Empathic concern	2.78	0.49	2.89	0.44	− 1.38	ns
Personal distress	3.58	0.57	2.77	0.68	7.61	< .001
Perspective taking	3.67	0.41	3.66	0.55	0.17	ns
*Cognitive coping strategies:*						
Downward comparison	2.53	1.38	5.55	1.00	− 14.84	< .001
Cognitive restructuring	2.64	1.13	1.48	1.15	6.00	< .001
Resolution/acceptance	4.74	1.74	4.23	1.67	1.75	ns
Regret	2.10	1.47	1.07	1.14	4.66	< .001
Denial	1.40	1.17	0.46	0.65	5.92	< .001

*t*—Student test; *p*—significance level; *ns*—non significance

A diagnosis of secondary traumatic stress (STS) can be made based on the sensitivity and specificity of results; in this case, a score of 33 points was taken as the cut-off point, i.e., the threshold value. Regarding SPTG, a score of ≥ 7 sten is considered an indicator of high growth (Ogińska-Bulik & Juczyński, 2020). Based on these adopted cut-points for the tools measuring STS and SPTG, our findings suggest that the risk of STS was 22.9% for clergymen and only 8.6% for therapists, while the chance of a high level of SPTG was 61.4% for clergymen and 42.9% for therapists.

The intensification of the secondary traumatic changes was analyzed with regard to different aspects of empathy and cognitive coping strategies. Among the three aspects of empathy, personal distress was found to be significantly higher among the clergymen (*p* < 0.001). This aspect measures the level of personal suffering, anxiety and discomfort in response to the serious negative experiences, i.e., the suffering, of other people.

It is believed that the consequences of secondary exposure to trauma depend mainly on the cognitive processes involved in dealing with traumatic experience. As shown in Table 2, the two groups differ very significantly (*p* < 0.001) for all strategies, except for resolution/acceptance. Interestingly, while the downward comparison strategy prevails in therapists, the others are much common among the clergymen.

### Predictors of Secondary Traumatic Stress and Secondary Posttraumatic Growth

The main aim of the research was to confirm whether empathy and the cognitive processing of trauma, i.e., strategies for coping with stressful events caused by exposure to secondary trauma, determine the consequences of secondary exposure to trauma. The relationships between negative and positive posttraumatic change, and empathy and coping strategies are given in Table 3.

**Table 3:** Correlation coefficients between empathy, cognitive coping strategies and STS, SPTG

Variables	Clergymen (*N* = 70)		Therapists (*N* = 70)	
	STS	SPTG	STS	SPTG
*Empathy:*				
Empathic concern	.33**	.03	.36**	.28*
Personal distress	− .01	.30**	.45***	.08
Perspective taking	.07	.24*	.29**	.44***
*Cognitive coping strategies:*				
Downward comparison	.08	.30	.21**	.18
Cognitive restructuring	− .30**	.38***	.07	.49***
Resolution/acceptance	− .34**	.12	.07	.46***
Regret	.52***	.08	.42***	.17
Denial	− .21	− .08	.35**	.04

Significance level: * < .05; ** < .01; *** < .001

Much greater differentiation of dependencies can be seen between the two groups regarding the occurrence of secondary posttraumatic growth (SPTG). While the severity of SPTG is associated with only one aspect of empathy, i.e., personal distress, among the clergymen, it appears to be associated with two other aspects, in therapists: concern for empathy and perspective taking. Generally, positive changes are associated with adaptive coping strategies; however, these changes were only associated with cognitive restructuring in the case of the clergy, but cognitive restructuring and resolution /acceptance in therapists. These relationships are statistically very significant (*p* < 0.001).

As the phenomenon of multi-collinearity may occur when the independent variables are too strongly correlated with each other, the inter-correlations between the variables were checked separately, i.e., within the group of clergymen and within the therapists (Table 4). Among the clergymen, empathic concern was found to co-relate with perspective taking and with two non-adaptive coping strategies; in contrast, in the group of therapists, all three components of empathy correlate with each other, as do all three adaptive strategies of coping with stress.

**Table 4:** Correlations between explanatory variables in the study groups: data above (therapists) and below (clergymen) the diagonal line

	Variables	1	2	3	4	5	6	7	8
1	Empathic concern		.56***	.56***	.14	.13	.22	.29*	.00
2	Personal distress	− .00		.32**	.19	.08	.05	.37**	.24*
3	Perspective taking	.40***	.21		.03	.27*	.40***	.17	.17
4	Downward comparison	− .01	.11	.15		.41***	.42***	− .02	.34**
5	Cognitive restructuring	− .17	.01	− .22	.17		.55***	− .12	.12
6	Resolution/acceptance	− .11	.08	.23	.26*	.36**		.08	.00
7	Regret	.31**	.10	.13	.13	− .19	− .29*		.17
8	Denial	.37**	− .01	.13	.24*	.11	.19	.15	

Significance level: * < .05; ** < .01; *** < .001

As the two groups demonstrated such differences in correlation coefficients, separate regression analyses were conducted for clergymen and therapists. Four regression models were built, i.e., for STS and SPTG, separately for clergymen and therapists. Only the variables relating to three aspects of empathy and five strategies of coping with stress, i.e., those which statistically significantly correlated with STS or SPTG, were entered into the model. Because the three aspects of empathy correlate strongly with each other, the one most strongly associated with STS, i.e., emphatic concern in the clergymen group and personal distress in the therapist group, was entered into the regression model. The multi-collinearity was estimated by calculating two parameters for each of the predictors, i.e., the tolerance index and the VIF parameter. The basic assumptions regarding the use of multiple linear regression were satisfied. The tolerance index was within 0.51–0.90, and VIF value close to 1.

The results of the multiple regression analysis (stepwise regression) for STS prediction are given in Table 5, and those for SPTG prediction in Table 6. Both tables include the results of all the variables that remained in the final model. Variables whose index of determination (R^2^) explains less than 5.0% of the variance are considered insignificant.

**Table 5:** Predictors of secondary traumatic stress

Predictors	*R*^2^	Beta	*B*	*t*	*p*
*Clergymen*			ns		
Constant value			7.52	0.79	ns
Regret	.27	.43	1.36	4.03	< .001
Cognitive restructuring	.04	− .19	− 0.60	− 1.88	ns
Empathic concern	.02	.16	0.41	1.48	ns
*R*^2^ = .33; *F* (3,66) = 11.02; *p* < .001					
*Therapists*					
Constant value			− 6.76	-1.34	ns
Personal distress	.20	.29	0.73	2.66	< .001
Regret	.08	.27	0.94	2.56	< .01
Denial	.05	.24	1.07	2.78	< .05
*R*^2^ = .33; *F* (3,66) = 10.81; *p* < .001					

*R*^2^—coefficient of determination; *Beta*—standardized regression coefficient

*B*—unstandardized regression coefficient

**Table 6:** Predictors of secondary posttraumatic growth

Predictors	*R*^2^	*Beta*	*B*	*t*	*p*
*Clergymen*					
Constant value			-7.56	-0.72	ns
Cognitive restructuring	.14	.44	3.65	4.16	< .001
Perspective taking	.11	.28	6.54	2.64	< .01
Personal distress	.05	.23	3.89	2.24	< .05
*R*^2^ = .30; *F* (3,63) = 9.61; *p* < .001					
*Therapists*					
Constant value			-2.24	-0.27	ns
Cognitive restructuring	.24	.31	0.85	3.69	< .001
Perspective taking	.10	.29	6.59	2.71	< .01
Resolution/acceptance	.02	.17	0.42	1.37	ns
*R*^2^ = .36; *F* (3,66) = 12.36; *p* < .001					

The best predictor of the severity of STS symptoms among clergymen was found to be the use of the regret strategy, which plays the greatest role in explaining total variability (*R*^2^ = 27.0%). However, among the therapists, who are generally characterized by a lower intensity of STS than the clergymen, the main determinants were personal distress (*R*^2^ = 20.0%), and to a lesser extent, the coping strategies regret and denial; the Beta coefficient values are positive, indicating that with an increase in regret strategy or personal distress, the intensity of STS increases. Regret is a non-adaptive strategy of dealing with stressful events; however, personal distress measures the tendency to experience fear or discomfort in response to the severe negative experience of others (item from ESS: *What frightens me is being in a situation of emotional tension*).

The predictors of SPTG among clergymen are the cognitive restructuring strategy and two aspects of empathy (perspective taking and personal distress). These explain 30.0% of the total variance.

What is the value of the presented models? In a well-fitted regression model, it is assumed that any resulting residual errors in predicting the actual value of the dependent variable based on the created regression model are independent of each other; in other words, their distribution is random. This assumption can be estimated using the Durbin-Watson test to detect autocorrelation in the residuals from a regression analysis. The statistic was found to be 2.18 for STS and 2.04 for SPTG among the clergymen, and 2.33 (STS) and 2.29 (SPTG) among the therapists. Values from 2 to 4 indicate negative autocorrelation.

## Discussion

Clergymen have a long history of helping in times of grief, crises, and trauma. While mental health is the focus of qualified professionals, many believers turn to the clergy for support; indeed, it is part of their pastoral responsibility to help and care for those who have experienced trauma (Mathews, 2007).

It was found that every fifth priest helping trauma victims is exposed to the negative consequences of STS; this number is much higher than that found among therapists, who also deal with trauma victims (almost 9%). The regret strategy proved to be the best predictor of the severity of STS symptoms among clergymen. This suggests that the intensification of STS symptoms in this group is determined by a maladaptive, and thus ineffective, coping strategy in the form of grief; in such cases, the clergymen would seem to blame themselves, at least partially, for the pain and suffering of others, which can increase susceptibility to the occurrence of STS. However, on the other hand, such an experience of regret may inspire the search for answers to existential questions, including the meaning of life, and thus may facilitate the occurrence of SPTG, although to a lesser extent than positive cognitive strategies.

Meanwhile, the main determinant of STS in therapists is an aspect of empathy: personal distress, i.e., the tendency to experience fear or discomfort in response to the severe negative experience of others. This suggests that the main factor determining the occurrence of STS in therapists are aspects of personality, including empathy, especially its emotional aspect. Additionally, clergymen were found to differ significantly from therapists regarding the cognitive processing of trauma. In difficult situations, therapists use mainly a downward comparison strategy to present their situation in a better light.

It is worth noting that the two professions differ in their training. The therapists, typically with a background in psychology, have had more training in human behavioral mechanisms and competence in coping with stress. In turn, clergymen appear to present deficits in efficient coping with trauma experienced by others. This difference may account for the observed differences between clergymen and therapists.

The clergy surveyed in the present study serve as chaplains in hospitals, hospices, crisis intervention centers, prisons, and the army, where their role is to support and help people struggling with difficult living conditions; in such cases, their duties seem to be of a therapeutic nature. Similar tasks are performed by therapists working with people who have experienced trauma. Both professions require empathy associated with emotional co-expression, compassion, and concern for the other person, as well as an understanding of their feelings and point of view. Without empathy in the midfielder, there can be no STS or emotional resonance (Figley, 2002); in addition, there is little chance of a therapeutic change or effective help for people experiencing trauma. Thus arises the paradox of empathy: while empathy is necessary to increase work efficiency, it can be harmful and a source of secondary traumatization in the helper.

Numerous studies in the field of psychotraumatology have found trauma to be “Janus-faced,” as STS symptoms have been found to be accompanied by secondary posttraumatic growth (SPTG). Such positive changes were observed among the most clergymen (61.0%), with a much higher prevalence than among therapists (43.0%); these changes were particularly apparent regarding spirituality.

The use of a cognitive restructuring strategy and empathy in the form of perspective taking appeared to be the best predictors of SPTG among both clergymen and therapists. This strategy reflects the tendency to adopt spontaneously someone else's point of view in everyday situations. Such an ability to see positive sides to a traumatic experience and express cognitive empathy involves the conveyance of understanding; this approach allows emotional balance to be maintained and for positive change to be stimulated, while keeping affective distance.

### Practical Implications

The present study examines both the negative and positive effects of secondary exposure to trauma. Few publications have examined both STS and SPTG. Our results suggest that the occurrence of negative posttraumatic symptoms is somehow necessary for the occurrence of positive change. Clergymen, like many other professions helping trauma survivors, are not sufficiently aware of this natural but potentially harmful aspect of their role. Comparatively little research has addressed the intangible cost to the clergymen of exposure to secondary traumatization (Hendron et al., 2014). Pearlman and Saakvitne (1995) warn that therapists who ignore the long-term influence of their work on their physical and mental wellbeing, and who do not take care of themselves in this regard, are prone to harmful effects such as burnout and vicarious traumatization. Figley, a specialist in compassion fatigue, suggests adding to the Hippocratic Oath, *primum non nocere*, the second clause for caregivers: *first do no SELF harm*.

Support should be provided to clergymen involved in helping trauma survivors, people suffering from severe or incurable illnesses or personal tragedies; such support is particularly valuable, as they find it difficult to achieve support in their community. Despite their good intentions, those helping others may not be fully prepared to deal with secondary consequences of trauma. Any such provider of support should be provided access to training to increase their competences in this area, as their activities have a strong influence on maintaining mental health.

In general, clergymen do not have the psychological qualifications or therapeutic preparation that would formally authorize them to conduct therapy, counseling, interventions, or other relief activities. As such, they should be encouraged to supplement and deepen their competences to be able to better support those experiencing trauma.

### Limitations and Future Directions

Our research has several limitations. First, the research is of a cross-sectional nature and is not a representative sample for Catholic clergymen. However, when developing a research plan on the secondary traumatization of clergy, we expected difficulties in reaching this group; therefore, we used trusted intermediaries and first obtained the consent of the clergy superiors. When inviting clergy to participate in the study, we described the main goal, which was to try to identify the risk of secondary traumatization, and then to prepare tools that would allow for self-monitoring of this risk.

In total, we received 70 completed tools. Such a low amount may be due to the fact that the clergy appear generally reluctant to talk about their own professional experiences. Such behavior may be caused by a sense of shame related to emotions that are difficult to control (Hendron et al., 2014). Future studies should therefore employ different approaches to reach respondents; perhaps through organized national meetings of clergymen working in institutions such as hospitals or prisons. A lecture on the issues raised and possible consequences could help overcome resistance and encourage greater participation in the research.

In addition, our study only analyzed the use of cognitive strategies for coping with stress and did not include equally significant behavioral strategies. No other personality-related properties, such as self-efficacy, were considered, nor was the importance of perceived social support. Finally, the traumatic experiences of the participants themselves were not considered.

## Conclusion

The mental health of helpers would be better protected if, rather than living by the emotions of their patients or clients, they were encouraged to understand their charges and express their willingness to help. We believe that our research represents a step toward a better understanding of the consequences of secondary trauma acquired by clergy as part of their duties. A positive aspect of the research may be the finding that exposure to secondary traumatization is largely compensated by the occurrence of positive growth. Clergymen who help others provide a good example of how their activities positively affect their spiritual development and self-confidence, as well as their appreciation of life and acceptance of helping others.

Previous studies published on the cooperation of clergymen and psychologists indicate the need for the clergy to broaden their knowledge about mental health, crisis interventions, counseling, and psychological therapy. Unfortunately, poor communication in this area is often seen as an obstacle to better cooperation (Oppenheimer et al., 2004; Weaver et al., 1997).
